# Inflammatory Bowel Diseases Phenotype, *C. difficile* and NOD2 Genotype Are Associated with Shifts in Human Ileum Associated Microbial Composition

**DOI:** 10.1371/journal.pone.0026284

**Published:** 2012-06-13

**Authors:** Ellen Li, Christina M. Hamm, Ajay S. Gulati, R. Balfour Sartor, Hongyan Chen, Xiao Wu, Tianyi Zhang, F. James Rohlf, Wei Zhu, Chi Gu, Charles E. Robertson, Norman R. Pace, Edgar C. Boedeker, Noam Harpaz, Jeffrey Yuan, George M. Weinstock, Erica Sodergren, Daniel N. Frank

**Affiliations:** 1 Department of Medicine, Stony Brook University, Stony Brook, New York, United States of America; 2 Department of Microbiology and Molecular Genetics, Stony Brook University, Stony Brook, New York, United States of America; 3 Department of Applied Mathematics and Statistics, Stony Brook University, Stony Brook, New York, United States of America; 4 Department of Ecology and Evolution, Stony Brook University, Stony Brook, New York, United States of America; 5 Department of Molecular, Cellular and Developmental Biology, University of Colorado, Boulder, Colorado, United States of America; 6 Department of Pediatrics, University of North Carolina, Chapel Hill, North Carolina, United States of America; 7 Departments of Medicine, Microbiology and Immunology, University of North Carolina, Chapel Hill, North Carolina, United States of America; 8 Department of Medicine, University of New Mexico, Albuquerque, New Mexico, United States of America; 9 Department of Pathology, Mount Sinai School of Medicine, New York, New York, United States of America; 10 Department of Medicine, Washington University, St. Louis, Missouri, United States of America; 11 Genome Institute, Washington University, St. Louis, Missouri, United States of America; 12 Division of Biostatistics, Washington University, St. Louis, Missouri, United States of America; 13 Department of Medicine, University of Colorado Anschutz Medical Campus, Aurora, Colorado, United States of America; Charité-University Medicine Berlin, Germany

## Abstract

We tested the hypothesis that Crohn’s disease (CD)-related genetic polymorphisms involved in host innate immunity are associated with shifts in human ileum–associated microbial composition in a cross-sectional analysis of human ileal samples. Sanger sequencing of the bacterial 16S ribosomal RNA (rRNA) gene and 454 sequencing of 16S rRNA gene hypervariable regions (V1–V3 and V3–V5), were conducted on macroscopically *disease-unaffected* ileal biopsies collected from 52 ileal CD, 58 ulcerative colitis and 60 control patients without inflammatory bowel diseases (IBD) undergoing initial surgical resection. These subjects also were genotyped for the three major NOD2 risk alleles (Leu1007fs, R708W, G908R) and the ATG16L1 risk allele (T300A). The samples were linked to clinical metadata, including body mass index, smoking status and *Clostridia difficile* infection. The sequences were classified into seven phyla/subphyla categories using the Naïve Bayesian Classifier of the Ribosome Database Project. Centered log ratio transformation of six predominant categories was included as the dependent variable in the permutation based MANCOVA for the overall composition with stepwise variable selection. Polymerase chain reaction (PCR) assays were conducted to measure the relative frequencies of the *Clostridium coccoides – Eubacterium rectales* group and the *Faecalibacterium prausnitzii* spp. Empiric logit transformations of the relative frequencies of these two microbial groups were included in permutation-based ANCOVA. Regardless of sequencing method, IBD phenotype, *Clostridia difficile* and NOD2 genotype were selected as associated (FDR ≤0.05) with shifts in overall microbial composition. IBD phenotype and NOD2 genotype were also selected as associated with shifts in the relative frequency of the *C. coccoides* – *E. rectales* group. IBD phenotype, smoking and IBD medications were selected as associated with shifts in the relative frequency of *F. prausnitzii spp.* These results indicate that the effects of genetic and environmental factors on IBD are mediated at least in part by the enteric microbiota.

## Introduction

Abnormal host-microbial interactions and genetic susceptibility are implicated in the pathogenesis of inflammatory bowel diseases (IBD) [1]–[4]. Culture-independent microbiological technologies coupled with high-throughput DNA sequencing have revolutionized the scale, speed, and economics of microbial ecological studies. When applied to IBD, these technologies have uncovered alterations in human intestine-associated microbial compositions (“dysbiosis”) in IBD patients compared with controls [5]–[12]. To further investigate mechanisms and the biological and clinical significance of dysbiosis in IBD, we have begun integrating metagenomic and phenotype data with genotype and additional clinical metadata.

We focused on the three prevalent risk alleles of the nucleotide oligomerization domain 2 (NOD2; Leu1007fs, R702W, and G908R) and the ATG16L1 T300A genotype out of the ∼100 IBD related genotypes identified thus far, because these loci have been linked to host innate immunity, particularly Paneth cell function, and ileal Crohn’s disease (CD) phenotype [13]–[27]. We recently conducted an exploratory study that integrated NOD2 and ATG16L1 genotype data with a previously published 16S rRNA sequence dataset [5], [11]. This analysis revealed potential associations between alterations in intestine-associated microbial composition and respectively disease phenotype, NOD2 and ATG16L1 genotype. One limitation was that the samples from IBD patients were collected from two separate anatomic sites (ileum and colon), and from both grossly disease-affected and disease unaffected regions. The CD patients were heterogeneous with respect to disease location and included patients with both ileal and colonic disease. There is evidence that patients with isolated colonic CD have distinct genetic characteristics from patients with ileal CD [28]. Genetic associations for Crohn’s colitis patients overlap extensively with UC patients and differ from ileal CD patients [29]. For example, the relative frequency of patients with at least one of the three major NOD2 risk alleles is only 16% in Crohn’s colitis patients, approaching the frequency observed in non-IBD control subjects [30]. Subphenotyping CD patients with respect to disease location would therefore facilitate biological interpretation of integrating metagenomic data with genotype data [31]–[33]. Another limitation of the previous study was that relatively limited clinical metadata was available for assessing the effects of potentially confounding variables, such as obesity [34].

In the current study, 16S rRNA sequence analysis was conducted on the proximal margins of resected ileum collected from a larger independent set of subjects with and without inflammatory disease to test the hypothesis that these genes affect ileum-associated microbiota in grossly *disease-unaffected* regions of the ileum, In contrast to the previous study, the subjects in the current study were restricted to three disease phenotypes that were unlikely to overlap with respect to disease location: 1.) Ileal CD patients undergoing ileocolic resection; 2.) colitis patients (without ileal disease) undergoing total colectomy or proctocolectomy; and 3.) control non-IBD patients undergoing either initial right hemicolectomy or total colectomy. Patients with ileocolic anastomoses from previous surgeries were excluded, because increased reflux of colonic luminal contents could potentially impact ileal mucosal microbial profiles. The samples were also linked to far more extensive clinical metadata than those used in the previous exploratory analysis [5], [11].

Because the previous dataset we analyzed was generated by amplifying the entire 16S rRNA gene followed by Sanger sequencing, this methodology was also applied in the current study. However, to increase depth of coverage and corroborate results derived from Sanger sequencing datasets, we also performed 454 sequencing of two regions of the 16S rRNA gene (V1–V3 and V3–V5) using primers adopted by the ongoing Human Microbiome Project [35]–[38]. These three parallel datasets provide a unique opportunity for comparing the results of these three sequencing methods in a disease setting.

## Materials and Methods

### Patients and Acquisition of Macroscopically Disease-Unaffected Proximal Margin Ileal Tissue Samples

This study was approved by the Institutional Review Boards of Washington University-St. Louis and Stony Brook University. The diagnosis of CD or UC was made ultimately on the basis of pathological criteria (surgical resection specimen) [31]–[33]. Ileal CD patients undergoing ileocolic resection, colitis patients undergoing total colectomy and control non-IBD patients undergoing either right hemicolectomy or total colectomy were prospectively enrolled in a consecutive fashion by the Washington University Digestive Diseases Research Core Center Tissue Procurement Facility to donate surgically resected tissue samples and clinical information between April 2005 and February 2010. The clinical information and patient samples were stripped of all identifying information and assigned a patient code and sample code.

The ileal CD patients were predominantly those falling within the Montreal classification of ileal disease with or without cecal disease (L1) [30]. Based on post-operative pathological diagnosis of the resected colon, 47 patients were diagnosed with UC, 9 patients were diagnosed with Crohn’s colitis and 2 patients were diagnosed with indeterminate colitis. Fifty-eight percent of the control non-IBD patients underwent resection for benign colonic diseases (colonic inertia, diverticulosis, adenomas, etc.) and the remaining 42% underwent resection for primary colonic adenocarcinomas. Patients who were unwilling or unable to give informed consent were excluded. Patients who had undergone previous resection of ileum as evidence by the presence of an ileocolonic anastomosis were excluded from this study. The number of subjects (n = 170) included in this cross-sectional study was designed to exceed the number of subjects studied previously (n = 125) [5], [11].

A minimum of 4 biopsies were taken from the macroscopically disease unaffected proximal ileal margin of fresh pathological specimens using Radial Jaw4 large-capacity biopsy forceps (Boston Scientific, Natick, MA), immediately placed in RNA stabilization solution (RNAlater, Applied Biosystems/Ambion, Austin, TX) and archived at −80°C [39]. The designation of *disease-unaffected* was based on the macroscopic appearance of the mucosa and the surgical pathology report of the adjacent biopsies (“no histopathologic abnormality”). The samples were de-identified and linked to a detailed clinical database by a patient study code.

Information on potential confounding variables was obtained by reviewing the medical records, including the pathological report of the resected intestine by a gastroenterologist (EL). Preoperative mechanical bowel preparations were not routinely ordered, particularly for the IBD patients. Also, adherence to bowel preparations was quite varied among participating subjects. For this reason, preoperative bowel preparation was not included in the analysis. A smoker was defined as smoking ≥7 cigarettes a week for at least a year [40]–[42]. The body mass index (BMI) was recorded for all individuals [34]. Concomitant *Clostridium difficile* infection was recorded as the presence of a positive fecal *C. difficile* toxin [43], [44]. Most of these patients were treated with metronidazole or oral vancomycin [45]. All of the patients received intravenous antibiotic prophylaxis covering both aerobic and anerobic bacteria (e.g. ciprofloxacin and metronidazole, cefoxitin, cefotetan) within one hour of incision [46].

### Genotyping of NOD2 and ATG16L1 Single Nucleotide Polymorphisms (SNPs)

Each patient was genotyped for the three major NOD2 SNPs, Leu1007fsInsC (rs2066847, SNP13), R702W (rs206884, SNP8) and G908R (rs2066845, SNP12) and for the autophagy like ATG16L1T300A SNP (rs2241880) by direct sequencing, and/or by a TaqMan MGB (Applied Biosystems, Foster City, CA) genotyping platform using genomic DNA prepared from peripheral blood and/or tissue by the Sequenom Technology Core within the Washington University Division of Human Genetics.(http://hg.wustl.edu/info/Sequenom_description.html) as previously described [39]. Because some combinations of individual NOD2 risk alleles, ATG16L1 risk alleles, and disease phenotype were not sampled in this study, the three NOD2 risk alleles were combined to form two composite categories: 1) NOD2^NR^, subjects harboring none of the three risk alleles (i.e., NOD2^NR/NR^); or 2) NOD2^R^, subjects harboring at least one of the three risk alleles (i.e., NOD2^R/R^ + NOD2^R/NR^). The three ATG16L1 genotype categories were: 1.) ATG16L1^NR/NR^, no ATG16L1 risk allele; 2.) ATG16L1^R/NR^, a single ATG16L1 risk allele; 3.) ATG16L1^R/R^, two ATG16L1 risk alleles.

### Library Construction and 16S rRNA Sequence Analysis

Parallel sequence datasets were generated for each of the samples at the Genome Institute at Washington University as previously described by 1.) broad-range PCR amplification of bacterial rRNA genes and Sanger sequencing and 2.) 454 sequencing of two separate segments of the 16S rRNA gene that encode either the V1 and V3 (V1–V3) or V3, V4, and V5 (V3–V5) hypervariable regions (see Methods S1) [35]–[37]. Of note, the 454 sequencing primers used in this current study were identical to the primers employed for characterizing the microbial communities in healthy individuals at different body sites, including the gastrointestinal tract by the Human Microbiome Project (http://hmpdacc.org/). The analysis software used to process the sequencing data consisted of established function specific tools that are described in greater detail in Methods S1. All sequences were screened for fidelity to a 16S rRNA bacterial covariance model (CM) based on secondary structure using the Infernal software package and were checked for chimerism with ChimeraSlayer [37], [47]. Potentially chimeric sequences and sequences lacking high fidelity to the CM were removed from subsequent analysis. Patient DNA samples with less than 100 total screened sequences were excluded from the analysis.

The sequences were classified into seven phyla/subphyla categories using the Naïve Bayesian Classifier of the Ribosome Database Project as described in Methods S1 [5], [11]: The seven categories were 1) *Actinobacteria*, 2) *Bacteroidetes*, 3) *Firmicutes.* Clostridium Group IV, 4) *Firmicutes.* Clostridium Group XIVa, 5.) *Firmicutes.* Bacillus, 6.) *Proteobacteria*, and 7.) Other taxa. The subdivisions of the Firmicutes phyla were based on concordance between the RDP classifier and the Greengenes 16S rRNA phylogenetic schema [5], [11], [48]–[51]. Six of these seven bacterial categories (Actinobacteria, Bacteroidetes, Firmicutes/Clostridium GroupIV, Firmicutes/Clostridium GroupXIVa, Firmicutes/Bacillus, and Proteobacteria) were selected as representing the overall microbial composition.

Assembled Sanger sequences were deposited in GenBank accession HQ739096-HQ821395. 454 V1–V3 and V3–V5 sequences were deposited in the Sequence Read Archive accession SRX021348-SRX021368, SRX037800-SRX037802. Clinical and genotyping data can be accessed through the dbGAP authorized access system. Request access to: phs000255. The study accession is SRP002479 “Effect of Crohn’s disease risk alleles on enteric microbiota”. In order to request access to any of the individual-level datasets within the controlled-access portions of the database, the Principal Investigator (PI) and the Signing Official (SO) at the investigator’s institution will need to co-sign a request for data access, which will be reviewed by an NIH Data Access Committee at the appropriate NIH Institute or Center https://dbgap.ncbi.nlm.nih.gov/aa/wga.cgi?page=login.

### Quantitative PCR (qPCR)

QPCR assays were performed for the *Clostridium coccoides* – *Eubacterium rectales Faecalibacterium prausnitzii* and total bacteria using established primers (see Methods S1) [52], [53]. The assays were carried out in triplicate. Plasmid quantification standards were prepared from representative clones of the target organisms.

### Statistical Analysis

Genotype and clinical categorical variables (e.g. disease phenotype, smoking, etc.) were compared between disease phenotypes using chi-square test for contingency tables. Clinical continuous variables, such as age and body mass index (BMI) were compared between disease phenotypes using the Kruskal-Wallis test. The relative frequencies of six of seven (excluding Other Taxa) categories selected to represent the overall microbial composition, were adjusted by adding 0.5 to all raw sequence counts in order to avoid 0% frequencies, and then subjected to centered log ratio transformation for the analysis of compositional data (see Methods S1) [54]. The effect of the independent variables and all first order interactions on these six bacterial categories (represented as a single vector), was analyzed in parallel for each sequencing platform by permutation based multivariate analysis of covariance (MANCOVA) with stepwise variable selection using the adonis function in R software (Version 2.12.1) package vegan (Version 1.17-2), Euclidean distances and a threshold significance level of 0.05 [55], [56]. To address the multiple comparison issue, we applied the Benjamini-Hochberg method to adjust P-values to the false discovery rate (FDR) [57]. The effect of these independent variables and their first order interactions on individual bacteria categories was further analyzed by permutation based analysis of covariance (ANCOVA) with stepwise variable selection and a threshold significance of 0.05 [32], [55], [56]. Repeated measures ANCOVA was then used to assess the union of the variables and first order interactions selected by the parallel ANCOVAs conducted on the three sequencing platforms separately, in an effort to utilize all three data sets simultaneously [58], [59]. The empirical logit transformations of the relative frequencies of the *C. coccoides – E. rectales* and the *F. prausnitzii* groups (measured by targeted qPCR) was used in the permutation-based ANCOVA. The Benjamini-Hochberg method was used to adjust P-values to the false discovery rate (FDR) [57]. The R codes are provided in Methods S1.

## Results

### Distribution of NOD2 and ATG16L1 Genotypes and Clinical Characteristics of Ileal CD, Colitis, and Non-IBD Control Subjects

As shown in Table 1, with the exception of race and gender, there were differences (FDR ≤0.05) in the distribution of the 11 remaining variables between the three disease phenotypes. For example, subjects who harbored at least one NOD2 risk allele, NOD2^R^, were more prevalent among ileal CD patients [16], [17], [39]. Only two (4%) ileal CD patients were ATG16L1^NR/NR^ Ileal CD patients were younger than the control patients at the time of surgery [23]. Actively smoking subjects were less prevalent in colitis patients [39]–[41]. The median BMI and age were lower in ileal CD subjects. *C. difficile* was more prevalent among subjects with colitis [43], [45]. None of the control subjects were treated with5-ASA, immunomodulators, and/or anti-TNFα biologics. All of the patients received intravenous antibiotic prophylaxis that covered both aerobic and anaerobic bacteria within one hour prior to incision [46].

**Table 1:** Distribution of NOD2 composite and ATG16L1 genotype and clinical characteristics of ileal CD, colitis and control non-IBD patients.

Variables	Ileal CD *(n = 52)*	Colitis *(n = 58)*	Control *(n = 60)*	P-value	FDR
**NOD2^R^ (R/R + R/NR)**	38%	15%	12%	**0.003**	**0.004**
**ATG16L1T300A (NR/NR)**	4%	28%	23%	**0.006**	**0.007**
Gender (male)	48%	57%	38%	0.130	0.14
Race (Caucasian)	92%	90%	83%	0.316	0.32
**Median age (range) y**	33 (18–72)	42 (17–68)	60 (32–64)	**<0.001**	**<0.001**
**Current smoker**	33%	3%	25%	**0.003**	**0.004**
**Positive fecal ** ***C. difficile*** ** toxin**	6%	28%	0%	**<0.001**	**<0.001**
**Median BMI (range) kg/m^2^**	24 (16–41)	25 (16–45)	28 (18–47)	**0.006**	**0.007**
**5-ASA**	52%	59%	0%	**<0.001**	**<0.001**
**Steroids**	54%	72%	0%	**<0.001**	**<0.001**
**Immunomodulators**	44%	72%	0%	**<0.001**	**<0.001**
**Anti-TNFα biologics**				**<0.001**	**<0.001**
Current (≤8 weeks of surgery)	29%	29%	0%		
Past (>8 weeks of surgery)	6%	7%	0%		
Never	65%	64%	0%		

The variables shown above are included in the subsequent MANCOVA and ANCOVA analyses. Chi-square test for contingency table was used for categorical data and Kruskal-Wallis test was used for age and BMI. To address multiple comparison issues, the Benjamini-Hochberg method was applied to adjust P-values to the false discovery rate (FDR).

### Comparison of the Relative Frequencies of Phyla/Subphyla Bacterial Categories between Ileal CD, Colitis and Control *Disease-Unaffected* Ileal Samples

**Figure 1:**
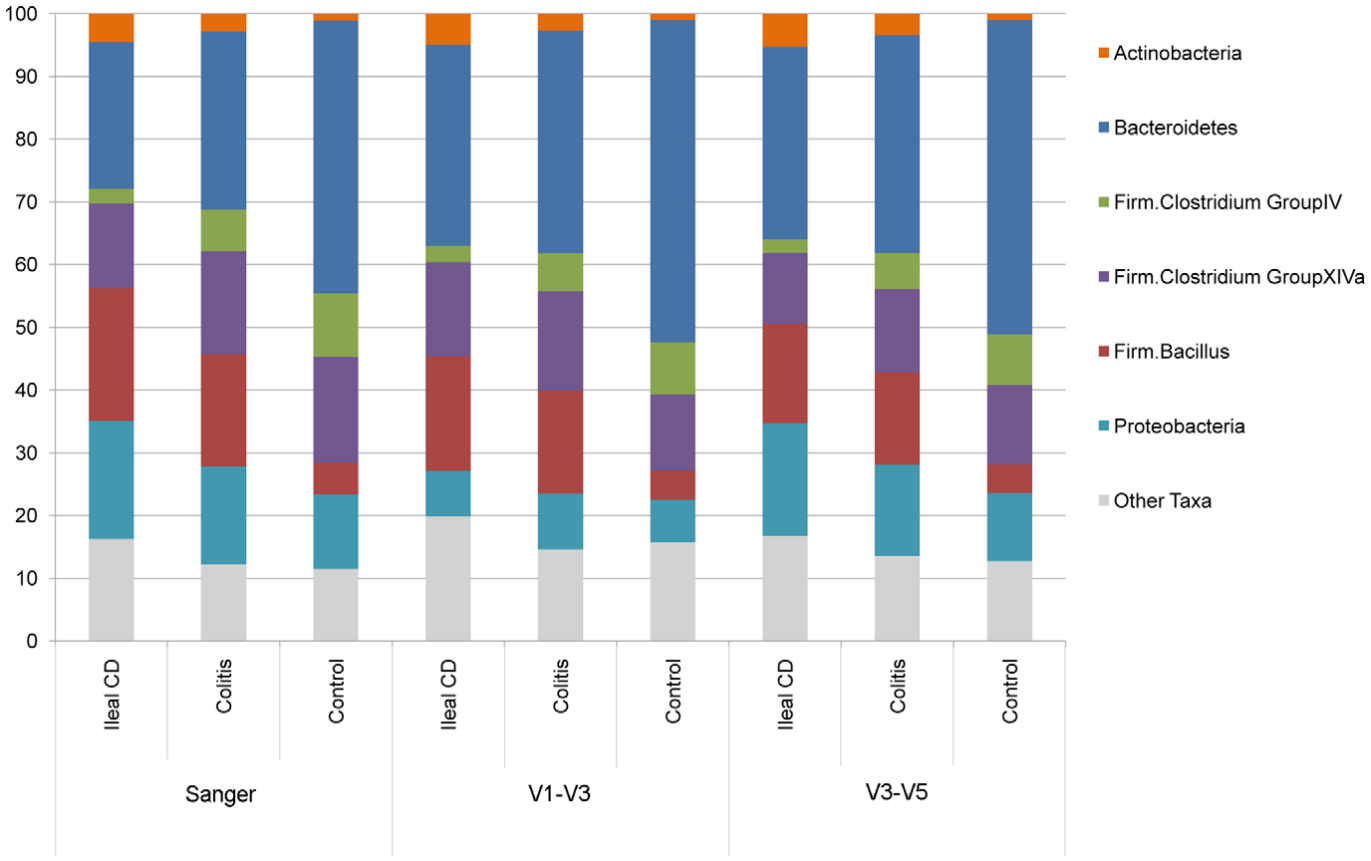
Phyla/subphyla comparison of three disease phenotypes (ileal CD, colitis, using the Sanger, 454 V1–V3 and 454 V3–V5 data sets. The average relative frequency of each taxa is shown for ileal CD, colitis and control subjects for each of the three sequencing data sets, (see also Table S1 for means ± standard deviations).

Using the Sanger method, a total of 81,644 near full length 16S rRNA sequences with acceptable quality were obtained with an average of 500 reads/sample. A total of 1,191,278 454 V1–V3 sequences (mean 7260 reads/sample) and a total of 917,900 454 V3–V5 sequences (mean 5400 reads/sample) were obtained from *disease-unaffected* samples. Greater than 90% of the sequences were binned into six of seven phyla/subphyla categories as shown in Figure 1. The Clostridium Group IV and Group XIVa taxa correspond to two prominent subsets of the “*Lachnospiraceae”* taxonomic group previously discussed by Frank et al. [5], [11]. As shown in Figure 1, and Table S1, the distribution of relative frequencies of the phyla/subphyla bacterial categories between the three disease phenotypes (ileal CD, colitis, control non-IBD) were very similar between the three datasets.

### Disease Phenotype, *C. difficile* and NOD2 Genotype are Associated with Shifts in Overall Disease-Unaffected Ileum-Associated Microbial Composition (MANCOVA)

The three sequence datasets were analyzed in parallel using a vector that combined the relative iesof the six most prevalent of the seven phyla/subphyla categories (see Figure 1) as the dependent variable. Exploratory permutation based MANCOVA with stepwise variable selection including all first order interactions, was conducted with each of the datasets. The parallel analyses with each dataset selected disease phenotype among 13 variables, as associated (FDR ≤0.05) with overall changes in the composition of mucosal bacterial communities (see Table 2). In addition, *C. difficile* and NOD2 genotype were selected (FDR ≤0.05) as associated with shifts in microbial composition. These two variables had smaller effects (R^2^) compared to the effect size of disease phenotype. Repeating the analysis with the 150 Caucasian subjects yielded similar results (see Table S2). Repeating the analysis after excluding subjects diagnosed with Crohn’s colitis and indeterminate colitis also yielded similar results (see Table S3).

**Table 2:** Permutation based MANCOVA with stepwise variable selection results for Sanger, 454 V1–V3 and 454 V3–V5 sequencing.

Sequencing	Sanger (*n = 164*)	R^2^	P value	FDR
**Main effects**	Disease phenotype	0.151	0.001	0.008
	*C. difficile*	0.019	0.019	0.04
	NOD2	0.018	0.011	0.03
**Interactions**	Disease phenotype * 5-ASA	0.022	0.012	0.03
	Steroids * Immunomodulators	0.022	0.006	0.03
	Disease phenotype * Age	0.033	0.008	0.03
**Sequencing**	**454 V1–V3** (*n = 164*)	**R^2^**	**P value**	**FDR**
**Main effects**	Disease phenotype	0.126	0.001	0.008
	*C. difficile*	0.019	0.016	0.03
	NOD2	0.017	0.029	0.05
**Interactions**	Disease phenotype * 5-ASA	0.022	0.011	0.03
	Steroids * Immunomodulators	0.022	0.006	0.03
	Disease phenotype * Age	0.032	0.009	0.03
**Sequencing**	**454 V3–V5** (*n = 169*)	**R^2^**	**P value**	**FDR**
**Main effects**	Disease phenotype	0.119	0.001	0.008
	*C. difficile*	0.020	0.014	0.03
	NOD2	0.029	0.004	0.02
**Interactions**	Disease phenotype * 5-ASA	0.020	0.015	0.03
	Steroids * Immunomodulators	0.030	0.001	0.008
	NOD2 * ATG16L1	0.024	0.028	0.05
	5-ASA * ATG16L1	0.024	0.040	0.06

The dependent variable was the vector generated by the centered log ratio of the relative frequencies of six phyla/subphyla categories (see text). The significant main effects and first order interactions selected by analysis of each of the three data sets as well as the R^2^, P values are listed here. To address multiple comparison issues, the Benjamini-Hochberg method was applied to adjust P-values to the false discovery rate (FDR). The number of samples (total = 170 samples) that yielded results suitable for analysis is listed for each method.

### Disease Phenotype Is Associated with Shifts in the Relative Frequencies of Actinobacteria, Bacteroidetes, Firmicutes. Clostridium GroupIV and Firmicutes. Bacillus Categories

In order to explore whether these variables were associated with particular microbial groups, permutation based ANCOVA with stepwise variable selection was carried out in parallel for each of the three datasets for each of the six phyla/subphyla categories (see Table S4). The union of the significant independent variables and first order interactions identified by these parallel analyses was then reanalyzed by permutation based repeated measures ANCOVA, in which the data from each sequence dataset was treated as a repeated measure (see Table 3). Analyzing the three datasets in parallel and as repeated measures (See Table 3 and Table S4), disease phenotype was selected as associated (FDR ≤0.05) with shifts in the relative frequencies of four of the six phyla/subphyla categories (Actinobacteria, Bacteroidetes, Firmicutes. Clostridium GroupIV and Firmicutes.Bacillus). Repeated measures ANCOVA also provided a means of comparing the results of the three sequencing methods. The three sequencing methods demonstrated good agreement (FDR>0.05) for the Actinobacteria and Bacteroidetes categories. Differences between the three sequencing methods may have the biggest effect (R^2^ = 0.040) on assessing the relative frequency of Proteobacteria.

**Table 3:** Permutation-based repeated measures ANCOVA results for each of the six phyla/subphyla bacterial categories (Clade).

Category/Clade	ACTINOBACTERIA	R^2^	P value	FDR
**Main effects**	**Disease phenotype**	**0.126**	**0.001**	**0.01**
	Steroids	0.021	0.029	0.08
**Interactions**	Age of Surgery * ATG16L1	0.041	0.014	0.07
**Measurements**		0.001	0.328	0.47

**Category/Clade**	**BACTEROIDETES**	**R^2^**	**P value**	**FDR**
**Main effects**	**Disease phenotype**	**0.117**	**0.001**	**0.01**
	Smoking	0.018	0.034	0.09
	5-ASA	0.021	0.025	0.08
	Steroids	0.023	0.013	0.07
**Interactions**	Steroids * Immunomodulators	**0.040**	**0.006**	**0.04**
	Disease phenotype * 5-ASA	0.019	0.023	0.08
	5-ASA * Age	0.016	0.050	0.12
	5-ASA * *C. difficile*	0.019	0.023	0.08
**Measurements**		0.002	0.047	0.12
**Category/Clade**	**FIRM.CLOSTRIDIUM GROUPIV**	**R^2^**	**P value**	**FDR**
**Main effects**	**Disease phenotype**	**0.161**	**0.001**	**0.01**
	**Gender**	**0.038**	**0.003**	**0.02**
	Smoking	0.025	0.015	0.07
	NOD2	0.021	0.027	0.08
**Interactions**	Disease phenotype * Age	**0.040**	**0.003**	**0.02**
	BMI * 5-ASA	0.025	0.010	0.06
**Measurements**		0.008	0.001	0.01
**Category/Clade**	**FIRM.CLOSTRIDIUM** **GROUPXIVa**	**R^2^**	**P value**	**FDR**
**Main effects**	Gender	0.023	0.019	0.08
**Interactions**	Disease phenotype * Age	**0.049**	**0.004**	**0.03**
	Steroids * BMI	0.030	0.011	0.06
**Measurements**		0.006	0.006	0.04
**Category/Clade**	**FIRM.BACILLUS**	**R^2^**	**P value**	**FDR**
**Main effects**	**Disease phenotype**	**0.175**	**0.001**	**0.01**
	NOD2	0.016	0.046	0.12
**Interactions**	**Disease phenotype** * Steroids	**0.040**	**0.002**	**0.02**
	Disease phenotype * Age	0.027	0.032	0.09
	5-ASA * ATG16L1	0.025	0.025	0.08
	Steroids * Immunomodulators	0.019	0.027	0.08
**Measurements**		0.008	0.001	0.01
**Category/Clade**	**PROTEOBACTERIA**	**R^2^**	**P value**	**FDR**
**Main effects**	NOD2	0.027	0.016	0.07
**Interactions**	Steroids * NOD2	**0.040**	**0.002**	**0.02**
	5-ASA * Race	**0.032**	**0.007**	**0.04**
	Steroids * Immunomodulators	0.021	0.030	0.08
	NOD2 * ATG16L1	0.056	0.019	0.08
**Measurements**		0.040	0.001	0.01

Sequencing results for all three platforms were available for 157 samples (44 ileal CD, 53 colitis and 60 control non-IBD). The variables and first order interactions with P≤0.05 are listed above. To address multiple comparison issues, the Benjamini-Hochberg method was applied to adjust P-values to the false discovery rate (FDR). The variables and first order interactions with FDR ≤0.05 are bolded.

### Quantification of Clostridium Coccoides – Eubacterium Rectales and Fecalibacterium Prausnitzii by qPCR

**Figure 2:**
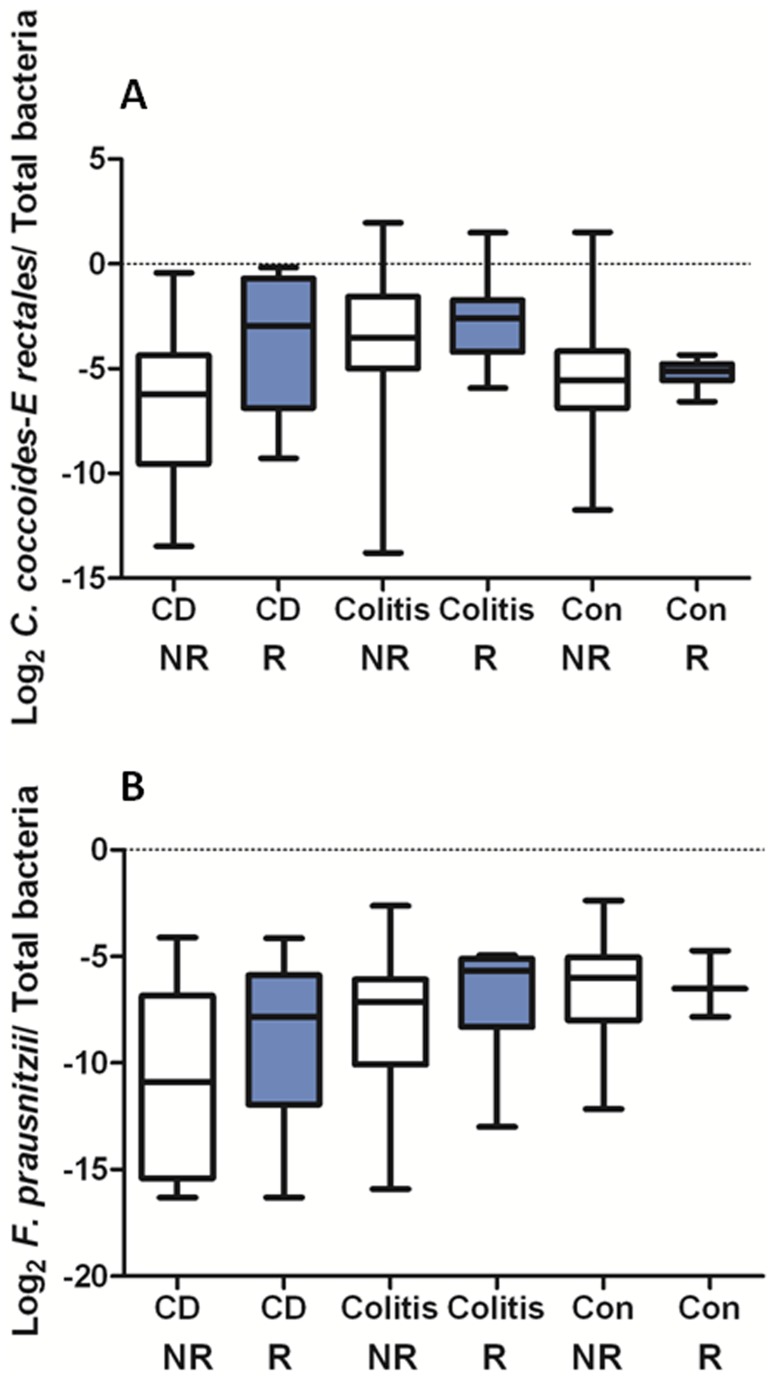
Targeted qPCR results for the *C. coccoides-E. rectales* group and for *F. prausnitzii* spp. Boxplots of (panel A) the log_2_ *C. coccoides-E. rectales* group/total bacteria and (panel B) the log_2_ *F. prausnitzii*/total bacteria as a function of disease phenotype and NOD2 genotype assayed using qPCR are shown. The middle line represents the median, and the lower edge and the upper edge of the box represent the 25% and 75% quartiles. The bottom and top lines represent the minimum and maximum values, respectively. For the *C. coccoides-rectales* group, all 170 samples were assayed. For *F. prausnitzii*, 157 of 170 samples were assayed.

QPCR analyses were conducted on total bacteria, the *C. coccoides-E. rectales* group and *F. prausnitzii* using previously established primers (see Figure 2).^52–53^ The relative frequency of the *C. coccoides-E. rectales* group, which overlaps the “*Lachnospiraceae”* taxonomic group (Clostridium GroupIV and XIVa are prominent subsets) was previously shown to be reduced in a subset of IBD subjects.^5^ *F. prausnitzii* is a major species within the Clostridium Group IV category. Low relative frequency of *F. prausnitzii* has been reported to be reduced in patients with ileal CD and has been associated with an increased risk of ileocolonoscopic recurrence of ileal CD [8], [9], [12].

As shown in Figure 2, the relative frequency of the *C. coccoides-E. rectales* group was significantly higher in NOD2^R^ ileal CD subjects than in NOD2^NR^ ileal CD subjects. In contrast, the relative frequency of the *C. coccoides-E. rectales* group was lower in ileal CD subjects compared to control non-IBD subjects, consistent with previous analysis of an independent set of tissues [5]. As shown in Table 4, IBD phenotype, NOD2 genotype and IBD medications were selected by ANCOVA with stepwise variable selection as associated (FDR ≤0.05) with shifts in the relative frequency of *C. coccoides-E. rectales* group. First order interactions between IBD phenotype and race, and between NOD2 genotype and steroids, were also selected. Unfortunately 13 DNA samples were exhausted before the qPCR assays for *F. prausnitzii* were conducted, reducing the total samples analyzed from 170 to 157. IBD phenotype, smoking and steroids were selected by ANCOVA with stepwise variable selection as associated (FDR ≤0.05) with shifts in the relative frequency of *F. prausnitzii* (see Table 4). First order interactions between NOD2 and smoking, and between age and gender were also selected.

**Table 4:** ANCOVA with stepwise variable selection results for relative frequencies of the *C. coccoides-E. rectales* microbial group and *F. prausnitzii* spp. based on targeted qPCR assays.

Category	*C. coccoides-E. rectales* n = 170	R^2^	P value	FDR
**Main effects**	**Disease phenotype**	**0.06368**	**0.001**	**0.006**
	**NOD2 genotype**	**0.08250**	**0.001**	**0.006**
	**Anti-TNFα**	**0.08216**	**0.001**	**0.006**
	**ASA**	**0.02904**	**0.009**	**0.02**
	**Immunomodulator**	**0.02548**	**0.019**	**0.03**
	Race	0.01050	0.125	0.16
	Steroids	0.00002	0.954	0.95
**Interactions**	Disease phenotype * Race	**0.05563**	**0.003**	**0.01**
	NOD2 * steroids	**0.02605**	**0.056**	**0.03**
**Category**	***F. prausnitzii*** n = 157	**R^2^**	**P value**	**FDR**
**Main effects**	**Disease phenotype**	**0.0662**	**0.002**	**0.008**
	**Smoking**	**0.0284**	**0.018**	**0.03**
	**Steroids**	**0.0270**	**0.017**	**0.03**
	NOD2	0.0033	0.394	0.48
	Age	0.0020	0.500	0.51
	Gender	0.0025	0.456	0.52
**Interactions**	Smoking * NOD2	**0.0528**	**0.004**	**0.01**
	Age * Gender	**0.0274**	**0.019**	**0.03**

See Materials and Methods. The variables and first order interactions with significant P values (≤0.05) as well as the variables in the first order interactions are listed above. To address multiple comparison issues, the Benjamini-Hochberg method was applied to adjust P-values to the false discovery rate (FDR). The variables and first order interactions with FDR ≤0.05 are bolded.

### Comparison of Non-IBD Control Samples from Subjects with and without Primary Colon Adenocarcinoma

Because a major proportion (42%) of the non-IBD control subjects underwent surgery for resection of right sided primary colon adenocarcinoma, we performed exploratory analyses comparing the relative frequencies of the seven phyla/subphyla clades as well as other clinical variables. As shown in Table S5, the only variable that was different (FDR ≤0.05) between the two groups of patients was the age of surgery, which is consistent with the observation that the incidence of colon cancer increases with patient age [60].

## Discussion

In this study we report the results of multiple regression analysis of the largest 16S rRNA sequence datasets reported thus far on ileal tissues collected from IBD subjects. This analysis demonstrates that IBD phenotype has a predominant effect on microbial composition associated with the macroscopically normal appearing proximal margin of resected ileum. The changes in intestinal microbiota that were observed in ileal CD may have occurred early in the pathogenic process before overt disease was manifest. Alternatively, chronic enteric dysbiosis that arises as a consequence of pathologic inflammation at one location within the GI tract may be propagated to unaffected sites. The observed alterations in ileal microbiota could have an impact on regional inflammation or metabolic properties (e.g., butyrate metabolism), however the functional implications of these shifts remain to be determined.

NOD2 genotype was also selected albeit with more modest effect for all three 16S rRNA sequence datasets and for targeted qPCR assays of the *C. coccoides- E. rectales* group. The bacterial 16S rRNA sequences assayed by the *C. coccoides – E. rectales* qPCR assay likely overlap with some but not all the 16S rRNA sequences included in Group XIVa and IV. The observation that ileal CD phenotype and NOD2 risk alleles, which presumably contribute to this phenotype, had opposite effects on this microbial group, suggests that the effect of the NOD2 risk allele on the relative frequency of the *C. coccoides-E. rectales* group is not simply mediated through an association with ileal CD phenotype. Thus the results of targeted QPCR assays and 16S rRNA sequence analysis both demonstrate a significant effect of NOD2 genotype on ileum associated microbial composition. Our results in disease –unaffected ileal tissues support our previous analysis of an independent set of intestinal tissues that were more heterogeneous with respect to anatomic location and inflammation. Our findings are consistent with the report that the relative frequency of *Firmicutes* spp., as determined by targeted qPCR, is higher in NOD2 knockout mice than in wild type mice [20], [21]. However, it is important to note that the patient-based studies are not directly comparable to the mouse studies because 1) the NOD2 knockout mouse may differ phenotypically from the NOD2Leu1007fs knock-in mouse [61], and 2) only 4% of the ileal CD patients compared to 28% of colitis and 23% of control non-IBD patients, were homozygous for the ATG16L1 T300A nonrisk allele (ATG16L1^NR/NR^). These findings are also consistent with the recent report that the relative frequency of Firmicutes measured by targeted qPCR is higher in three CD patients that were homozygous for the Leu1007fs (SNP13) compared to 11 CD patients that were homozygous for the wild type allele [21]. Since only two of the ileal CD patients were homozygous for the Leu1007fs allele in our dataset, a parallel comparison could not be made (see Table S6).

*C. difficile* infections, particularly recurrent *C. difficile* infections have been associated with altered fecal microbial composition [62], [63]. Patients with inflammatory bowel diseases are more likely to develop *C. difficile* infections, which are also associated with clinical exacerbation of their disease [43], [44]. It is possible that the shifts in microbial composition associated with IBD contribute to these patients’ susceptibility to *C. difficile* and other infections that may exacerbate inflammation. Alternatively antibiotic treatment of subjects with *C. difficile* could contribute to the observed shifts in microbial composition [64], [65].

Sanger sequencing of the entire 16S rRNA gene permits accurate phylogenetic identification of bacteria, whereas 454 pyrosequencing generates much greater depth of coverage, but with lesser phylogenetic resolution. While the results of all three sequencing methodologies demonstrated associations of disease phenotype, *C. difficile* infection and NOD2 genotype to the overall microbial composition in parallel analyses, they differed with respect to the main effects and first order interactions associated with individual phyla/subphyla categories. Methodological differences may reflect biases introduced during the initial PCR amplification step, as noted by Olsen and coworkers for the 27F forward primer [66]. In addition, there are likely differences in the phylogenetic resolution/assignment of the complete 16S sequence as opposed to different hypervariable regions (V1–V3 and V3–V5) of the gene sequence. Although the results from each sequencing method may converge with increasing the sample size, differences in the microbial composition data generated by different sequencing methods will make it challenging to compare results from studies using different primers for the initial PCR amplification.

The *F. prausnitzii* 16 S rRNA sequences assayed by targeted qPCR form a major subset of all the sequences grouped within the Firmicutes.Clostridium Group IV clade. The selection of smoking as a potentially significant covariate is intriguing, since smoking has been associated with ileal CD phenotype and with early postoperative recurrence in ileal CD patients, and is consistent with the observations of Sokol and coworkers that low ileal mucosal concentrations of *F. prausnitzii* is associated with early postoperative ileocolonoscopic recurrence of CD [8], [34]–[42]. Smoking was also selected by analysis of the Sanger and 454V1–V3 datasets as significantly associated with shifts in relative frequency of the Firmicutes.Clostridium Group IV clade (Table S4). Smoking cessation has clearly been linked to altering the subgingival microbial profile [67], but has not been previously linked to altering the ileum associated microbial profile.

Although pathologic review of the resected tissues provided rigorous phenotyping of the samples, the use of surgically resected tissues may bias the results by sampling of IBD patients with relatively severe disease who have been treated for various lengths of time with antibiotics and different IBD medications. Although the focus of this study was ileal CD, it is likely that other human diseases will exhibit similar links between genetically determined defects in mucosal immunity and alterations of resident microbiota. As we further expand these datasets by analyzing more samples, we anticipate that further associations between microbial composition, IBD subphenotypes, IBD polymorphisms and environmental factors will emerge.

## Supporting Information

Table S1 **A. Relative frequencies of the six phyla/subphyla categories selected to represent overall microbial composition based on the Sanger dataset.** The mean value ± standard deviation is shown for each of the three disease phenotypes, ileal CD, colitis and control non-IBD. **B. Relative frequencies of the six phyla/subphyla categories selected to represent the overall microbial composition based on the 454 V1–V3 dataset.** The mean value ± standard deviation is shown for each of the three disease phenotypes, ileal CD, colitis and control non-IBD. **C. Relative frequencies of the six phyla/subphyla categories selected to represent the overall microbial composition based on the 454 V3–V5 dataset.** The mean value ± standard deviation is shown for each of the three disease phenotypes, ileal CD, colitis and control non-IBD.
(DOCX)

Table S2 **Permutation-based MANCOVA with stepwise variable selection results for Caucasian patients.** Because NOD2 risk alleles are rarely observed in subjects of Asian and African descent, the analysis was repeated for the 150 Caucasian subjects in the study (48 ileal CD, 52 colitis, 50 non-IBD control subjects). The dependent variable was the vector generated by the centered log ratio of the relative frequencies of six phyla/subphyla categories (see text). The significant main effects and first order interactions selected by analysis of each of the three data sets as well as the R^2^, P values are listed below. To address multiple comparison issues, the Benjamini-Hochberg method was applied to adjust P-values to the false discovery rate (FDR). The number of samples (total = 150 samples) that yielded results suitable for analysis is listed for each method.
(DOCX)

Table S3 **Permutation-based MANCOVA with stepwise variable selection results for Sanger, 454 V1–V3 and 454 V3–V5 sequencing.** Samples with Crohn’s colitis and indeterminate colitis were excluded in this analysis. The dependent variable was the vector generated by the centered log ratio of the relative frequencies of six phyla/subphyla categories (see text). The significant main effects and first order interactions selected by analysis of each of the three data sets as well as the R^2^, P values are listed below. To address multiple comparison issues, the Benjamini-Hochberg method was applied to adjust P-values to the false discovery rate (FDR). The number of samples (around 150 samples) that yielded results suitable for analysis is listed for each method.
(DOCX)

Table S4 **Permutation based ANCOVA with stepwise variable selection results for each of the six individual phyla/subphyla categories.** Permutation based ANCOVA with step wise variable selection was carried out for the individual phyla/subphyla categories. in parallel for each of the datasets. A total of 164 samples were analyzed for the Sanger and the 454 V1–V3 datasets respectively. A total of 169 samples were analyzed for the 454 V3–V5 dataset. Listed below are the main effects, first order interactions with P-values ≤0.05, as well as the main effects of first order interactions with P values ≤0.05. To address multiple comparison issues, the Benjamini-Hochberg method was applied to adjust P-values to the false discovery rate (FDR). The main effects and first order interactions with FDR ≤0.05 are bolded.
(DOCX)

Table S5 **Comparison between nonIBD control subjects with primary colon adenocarcinoma with non-IBD subjects without primary colon adenocarcinoma.** Continuous variables (e.g. age, BMI, relative frequency of bacterial groups) were compared using the Wilcoxon rank sum test and categorical variables (e.g. genotype, smoking, race) were compared using the chi-square test. Note that none of the non-IBD control subjects had a positive C. difficile toxin or were taking any IBD medications. To address multiple comparison issues, the Benjamini-Hochberg method was applied to adjust P-values to the false discovery rate (FDR). Variables with FDR ≤0.05 are bolded.
(DOCX)

Table S6 **Distribution of the three common NOD2 genotypes in ileal CD, colitis and non-IBD control subjects.** The three major NOD2 risk alleles that account for 80% of the NOD2 variants, are Leu1007fs (SNP13, rs2066847), R702W (SNP8, rs2066844), and G908W (SNP12, rs2066845).
(DOCX)

Methods S1 (DOC)
