# Supplementary material for: Inflammatory Bowel Diseases Phenotype, C. difficile and NOD2 Genotype Are Associated with Shifts in Human Ileum Associated Microbial Composition
Source: PLoS One. 2012 Jun 13;7(6):e26284. doi: 10.1371/journal.pone.0026284 (PMC3374607; doi:10.1371/journal.pone.0026284)
Supplement: Table S1 — A. Relative frequencies of the six phyla/subphyla categories selected to represent overall microbial composition based on the Sanger dataset. The mean value ± standard deviation is shown for each of the three disease phenotypes, ileal CD, colitis and control non-IBD. B. Relative frequencies of the six phyla/subphyla categories selected to represent the overall microbial composition based on the 454 V1–V3 dataset. The mean value ± standard deviation is shown for each of the three disease phenotypes, ileal CD, colitis and control non-IBD. C. Relative frequencies of the six phyla/subphyla categories selected to represent the overall microbial composition based on the 454 V3–V5 dataset. The mean value ± standard deviation is shown for each of the three disease phenotypes, ileal CD, colitis and control non-IBD. (DOCX) [file pone.0026284.s001.docx]

**Supplementary Table S1A. Relative frequency of the six phyla/subphyla categories selected to represent overall microbial composition based on the Sanger dataset.** The mean value ± standard deviation is shown for each of the three disease phenotypes, ileal CD, colitis and control non-IBD.

| **Phyla/subphyla** | **Ileal CD**  n = 51 | **Colitis**  n = 53 | **Control**  n = 60 |
| --- | --- | --- | --- |
| Actinobacteria | 0.045 ± 0.066 | 0.028 ± 0.058 | 0.011 ± 0.051 |
| Bacteroidetes | 0.233 ± 0.248 | 0.284 ± 0.220 | 0.434 ± 0.178 |
| Firmicutes.Clostridium GroupIV | 0.023 ± 0.035 | 0.066 ± 0.092 | 0.101 ± 0.084 |
| Firmicutes. Clostridium.GroupXIVa | 0.135 ± 0.141 | 0.164 ± 0.167 | 0.169 ± 0.103 |
| Firmicutes. Bacillus | 0.212 ± 0.233 | 0.179 ± 0.219 | 0.051 ± 0.110 |
| Proteobacteria | 0.188 ± 0.232 | 0.156 ± 0.201 | 0.119 ± 0.177 |

**Supplementary Table S1B. Relative frequency of the six phyla/subphyla categories selected to represent the overall microbial composition based on the 454 V1-V3 dataset.** The mean value ± standard deviation is shown for each of the three disease phenotypes, ileal CD, colitis and control non-IBD.

| **Phyla/subphyla** | **Ileal CD**  n = 46 | **Colitis**  n = 58 | **Control**  n = 60 |
| --- | --- | --- | --- |
| Actinobacteria | 0.050 ± 0.094 | 0.027 ± 0.044 | 0.010 ± 0.013 |
| Bacteroidetes | 0.320 ± 0.261 | 0.354 ± 0.214 | 0.513 ± 0.159 |
| Firmicutes.Clostridium GroupIV | 0.026 ± 0.034 | 0.061 ± 0.071 | 0.083 ± 0.070 |
| Firmicutes. Clostridium.GroupXIVa | 0.150 ± 0.149 | 0.157 ± 0.145 | 0.121 ± 0.062 |
| Firmicutes. Bacillus | 0.183 ± 0.224 | 0.165 ± 0.204 | 0.047 ± 0.085 |
| Proteobacteria | 0.072 ± 0.122 | 0.089 ± 0.145 | 0.068 ± 0.093 |

**Supplementary Table S1C. Relative frequency of the six phyla/subphyla categories selected to represent the overall microbial composition based on the 454 V3-V5 dataset.** The mean value ± standard deviation is shown for each of the three disease phenotypes, ileal CD, colitis and control non-IBD.

| **Phyla/subphyla** | **Ileal CD**  **n = 51** | **Colitis**  **n = 58** | **Control**  **n = 60** |
| --- | --- | --- | --- |
| Actinobacteria | 0.053 ± 0.076 | 0.034 ± 0.076 | 0.010 ± 0.020 |
| Bacteroidetes | 0.307 ± 0.264 | 0.347 ± 0.251 | 0.501 ± 0.185 |
| Firmicutes.Clostridium GroupIV | 0.021 ± 0.030 | 0.058 ± 0.069 | 0.081 ± 0.070 |
| Firmicutes. Clostridium.GroupXIVa | 0.113 ± 0.117 | 0.132 ± 0.136 | 0.126 ± 0.087 |
| Firmicutes. Bacillus | 0.159 ± 0.185 | 0.148 ± 0.197 | 0.045 ± 0.083 |
| Proteobacteria | 0.179 ± 0.204 | 0.145 ± 0.199 | 0.108 ± 0.174 |
