# Supplementary material for: Inflammatory Bowel Diseases Phenotype, C. difficile and NOD2 Genotype Are Associated with Shifts in Human Ileum Associated Microbial Composition
Source: PLoS One. 2012 Jun 13;7(6):e26284. doi: 10.1371/journal.pone.0026284 (PMC3374607; doi:10.1371/journal.pone.0026284)
Supplement: Table S2 — Permutation-based MANCOVA with stepwise variable selection results for Caucasian patients. Because NOD2 risk alleles are rarely observed in subjects of Asian and African descent, the analysis was repeated for the 150 Caucasian subjects in the study (48 ileal CD, 52 colitis, 50 non-IBD control subjects). The dependent variable was the vector generated by the centered log ratio of the relative frequencies of six phyla/subphyla categories (see text). The significant main effects and first order interactions selected by analysis of each of the three data sets as well as the R2, P values are listed below. To address multiple comparison issues, the Benjamini-Hochberg method was applied to adjust P-values to the false discovery rate (FDR). The number of samples (total = 150 samples) that yielded results suitable for analysis is listed for each method. (DOCX) [file pone.0026284.s002.docx]

**Supplementary Table S2. Permutation based MANCOVA with stepwise variable selection results for Caucasian patients.** Because NOD2 risk alleles are rarely observed in subjects of Asian and African descent, the analysis was repeated for the 150 Caucasian subjects in the study (48 ileal CD, 52 colitis, 50 non-IBD control subjects). The dependent variable was the vector generated by the centered log ratio of the relative frequency of six phyla/subphyla categories (see text). The significant main effects and first order interactions selected by analysis of each of the three data sets as well as the R^2^, P values are listed below. To address multiple comparison issues, the Benjamini-Hochberg method was applied to adjust P-values to the false discovery rate (FDR). The number of samples (total = 150 samples) that yielded results suitable for analysis is listed for each method.

| **Sequencing** | **Sanger** (n = 144) | **R^2^** | **P value** | **FDR** |
| --- | --- | --- | --- | --- |
| **Main effects** | Disease phenotype | 0.154 | 0.001 | 0.007 |
|  | *C. difficile* | 0.015 | 0.036 | 0.06 |
|  | NOD2 | 0.019 | 0.003 | 0.05 |
| **Interactions** | Disease phenotype * 5-ASA | 0.023 | 0.013 | 0.03 |
|  | Steroids * Immunomodulators | 0.032 | 0.005 | 0.03 |
|  | Disease phenotype * Age | 0.030 | 0.022 | 0.03 |
|  | | | | |
| **Sequencing** | **454 V1-V3** (n = 145) | **R^2^** | **P value** | **FDR** |
| **Main effects** | Disease phenotype | 0.102 | 0.001 | 0.007 |
|  | NOD2 | 0.023 | 0.006 | 0.02 |
|  | Gender | 0.018 | 0.026 | 0.05 |
| **Interactions** | Disease phenotype * 5-ASA | 0.008 | 0.004 | 0.02 |
|  | Steroids * Immunomodulators | 0.043 | 0.002 | 0.01 |
|  | 5-ASA * BMI | 0.029 | 0.005 | 0.02 |
|  | NOD2 * ATG16L1 | 0.039 | 0.001 | 0.02 |
|  | 5-ASA * ATG16L1 | 0.030 | 0.021 | 0.04 |
|  | | | | |
| **Sequencing** | **454 V3-V5** (n = 150) | **R^2^** | **P value** | **FDR** |
| **Main effects** | Disease phenotype | 0.098 | 0.001 | 0.007 |
|  | *C. difficile* | 0.024 | 0.013 | 0.03 |
|  | NOD2 | 0.030 | 0.005 | 0.02 |
| **Interactions** | Disease phenotype * 5-ASA | 0.027 | 0.006 | 0.02 |
|  | Steroids * Immunomodulators | 0.049 | 0.001 | 0.007 |
|  | NOD2 * ATG16L1 | 0.040 | 0.009 | 0.02 |
|  | 5-ASA * ATG16L1 | 0.028 | 0.030 | 0.05 |
