# Supplementary material for: Inflammatory Bowel Diseases Phenotype, C. difficile and NOD2 Genotype Are Associated with Shifts in Human Ileum Associated Microbial Composition
Source: PLoS One. 2012 Jun 13;7(6):e26284. doi: 10.1371/journal.pone.0026284 (PMC3374607; doi:10.1371/journal.pone.0026284)
Supplement: Table S4 — Permutation based ANCOVA with stepwise variable selection results for each of the six individual phyla/subphyla categories. Permutation based ANCOVA with step wise variable selection was carried out for the individual phyla/subphyla categories. in parallel for each of the datasets. A total of 164 samples were analyzed for the Sanger and the 454 V1–V3 datasets respectively. A total of 169 samples were analyzed for the 454 V3–V5 dataset. Listed below are the main effects, first order interactions with P-values ≤0.05, as well as the main effects of first order interactions with P values ≤0.05. To address multiple comparison issues, the Benjamini-Hochberg method was applied to adjust P-values to the false discovery rate (FDR). The main effects and first order interactions with FDR ≤0.05 are bolded. (DOCX) [file pone.0026284.s004.docx]

**SupplementaryTable S4. Permutation based ANCOVA with stepwise variable selection results for each of the six individual phyla/subphyla categories.**  Permutation based ANCOVA with step wise variable selection was carried out for the individual phyla/subphyla categories. in parallel for each of the datasets. A total of 164 samples were analyzed for the Sanger and the 454 V1-V3 datasets respectively. A total of 169 samples were analyzed for the 454 V3-V5 dataset. Listed below are the main effects, first order interactions with P-values ≤ 0.05, as well as the main effects of first order interactions with P values ≤ 0.05. To address multiple comparison issues, the Benjamini-Hochberg method was applied to adjust P-values to the false discovery rate (FDR). The main effects and first order interactions with FDR ≤ 0.05 are bolded.

| **Category** | **ACTINOBACTERIA** |  |  |  |
| --- | --- | --- | --- | --- |
| **Sequencing** | **Sanger** | **R^2^** | **P value** | **FDR** |
| Main effects | **Disease phenotype** | **0.145** | **0.001** | **0.01** |
|  | ***C. difficile*** | **0.038** | **0.009** | **0.04** |
|  | Steroids | 0.023 | 0.034 | 0.08 |
|  | NOD2 | 0.000 | 0.746 | 0.82 |
|  | BMI | 0.001 | 0.924 | 0.94 |
| Interactions | BMI * NOD2 | 0.021 | 0.045 | 0.08 |
|  |  |  |  |  |
| **Sequencing** | **454 V1-V3** | **R^2^** | **P value** | **FDR** |
| Main effects | **Disease phenotype** | **0.145** | **0.001** | **0.01** |
|  | ***C. difficile*** | **0.038** | **0.004** | **0.03** |
|  | Steroids | 0.023 | 0.039 | 0.08 |
|  | NOD2 | 0.000 | 0.756 | 0.82 |
|  | Smoking | 0.000 | 0.931 | 0.94 |
| Interactions | Smoking * NOD2 | 0.023 | 0.031 | 0.08 |
|  |  |  |  |  |
| **Sequencing** | **454 V3-V5** | **R^2^** | **P value** | **FDR** |
| Main effects | **Disease phenotype** | **0.140** | **0.001** | **0.01** |
|  | *C. difficile* | 0.015 | 0.067 | 0.11 |
|  | Steroids | 0.011 | 0.123 | 0.18 |
|  | NOD2 | 0.005 | 0.282 | 0.38 |
|  | Age | 0.016 | 0.071 | 0.11 |
|  | ATG16L1 | 0.004 | 0.618 | 0.70 |
|  | 5-ASA | 0.001 | 0.707 | 0.79 |
| Interactions | Age * ATG16L1 | 0.042 | 0.018 | 0.06 |
|  | 5-ASA * *C. difficile* | 0.022 | 0.019 | 0.06 |
|  | Steroids * NOD2 | 0.014 | 0.087 | 0.14 |
|  |  |  |  |  |
| **Category** | **BACTERIODETES** |  |  |  |
| **Sequencing** | **Sanger** | **R^2^** | **P value** | **FDR** |
| Main effects | **Disease phenotype** | **0.155** | **0.001** | **0.01** |
|  | 5-ASA | 0.022 | 0.029 | 0.08 |
|  | *C. difficile* | 0.016 | 0.062 | 0.10 |
|  | Immunomodulators | 0.000 | 0.907 | 0.93 |
|  | Gender | 0.007 | 0.220 | 0.30 |
|  | Smoking | 0.010 | 0.112 | 0.17 |
|  | NOD2 | 0.002 | 0.476 | 0.58 |
|  | Age | 0.002 | 0.476 | 0.58 |
| Interactions | Immunomodulators * Gender | 0.020 | 0.046 | 0.08 |
|  | **Smoking * NOD2** | **0.052** | **0.002** | **0.02** |
|  | Disease phenotype * ASA | 0.017 | 0.037 | 0.08 |
|  | 5-ASA * Age | 0.023 | 0.017 | 0.06 |
|  | 5-ASA * *C. difficile* | 0.021 | 0.035 | 0.08 |
|  |  |  |  |  |
| **Sequencing** | **454 V1-V3** | **R^2^** | **P value** | **FDR** |
| Main effects | **Disease phenotype** | **0.155** | **0.001** | **0.01** |
|  | 5-ASA | 0.022 | 0.02 | 0.06 |
|  | Steroids | 0.018 | 0.046 | 0.08 |
|  | *C. difficile* | 0.016 | 0.055 | 0.09 |
|  | Immunomodulators | 0.000 | 0.814 | 0.87 |
|  | Gender | 0.007 | 0.206 | 0.29 |
|  | Smoking | 0.009 | 0.164 | 0.24 |
|  | NOD2 | 0.003 | 0.368 | 0.48 |
|  | Age | 0.001 | 0.611 | 0.70 |
| Interactions | Immunomodulators * Gender | 0.023 | 0.019 | 0.06 |
|  | **Smoking * NOD2** | **0.043** | **0.002** | **0.02** |
|  | Disease phenotype *ASA | 0.024 | 0.023 | 0.07 |
|  | **5-ASA * Age** | **0.029** | **0.01** | **0.04** |
|  | *C. difficile* * ATG16L1 | 0.037 | 0.013 | 0.05 |
|  |  |  |  |  |
| **Sequencing** | **454 V3-V5** | **R^2^** | **P value** | **FDR** |
| Main effects | **Disease phenotype** | **0.131** | **0.001** | **0.01** |
|  | 5-ASA | 0.018 | 0.033 | 0.08 |
|  | Steroids | 0.018 | 0.048 | 0.08 |
|  | *C. difficile* | 0.015 | 0.048 | 0.08 |
|  | Immunomodulators | 0.016 | 0.046 | 0.08 |
|  | NOD2 | 0.015 | 0.067 | 0.11 |
| Interactions | **Steroids * Immunomodulators** | **0.063** | **0.001** | **0.01** |
|  | **Disease phenotype * ASA** | **0.038** | **0.006** | **0.03** |
|  | ASA * NOD2 | 0.019 | 0.036 | 0.08 |
|  |  |  |  |  |
| **Category** | **FIRM. CLOSTRIDIUM GROUP IV** |  |  |  |
| **Sequencing** | **Sanger** | **R^2^** | **P value** | **FDR** |
| Main effects | **Disease phenotype** | **0.241** | **0.001** | **0.01** |
|  | **Gender** | **0.033** | **0.006** | **0.03** |
|  | **Smoking** | **0.030** | **0.006** | **0.03** |
|  | *C. difficile* | 0.013 | 0.078 | 0.12 |
|  | NOD2 | 0.013 | 0.070 | 0.11 |
|  | Age | 0.002 | 0.456 | 0.57 |
| Interactions | **Disease phenotype * Age** | **0.046** | **0.005** | **0.03** |
|  | *C. difficile* * NOD2 | 0.019 | 0.029 | 0.08 |
|  |  |  |  |  |
| **Sequencing** | **454 V1-V3** | **R^2^** | **P value** | **FDR** |
| Main effects | **Disease phenotype** | **0.220** | **0.001** | **0.01** |
|  | **Gender** | **0.033** | **0.006** | **0.03** |
|  | **Smoking** | **0.030** | **0.007** | **0.03** |
|  | *C. difficile* | 0.013 | 0.071 | 0.11 |
|  | NOD2 | 0.013 | 0.074 | 0.12 |
|  | Age | 0.002 | 0.465 | 0.57 |
| Interactions | **Disease phenotype * Age** | **0.046** | **0.004** | **0.03** |
|  | *C. difficile* * NOD2 | 0.019 | 0.034 | 0.08 |
|  |  |  |  |  |
| **Sequencing** | **454 V3-V5** | **R^2^** | **P value** | **FDR** |
| Main effects | **Disease phenotype** | **0.181** | **0.001** | **0.01** |
|  | ***C. difficile*** | **0.032** | **0.005** | **0.03** |
|  | **NOD2** | **0.069** | **0.001** | **0.01** |
|  | Age | 0.000 | 0.845 | 0.89 |
|  | 5-ASA | 0.005 | 0.290 | 0.38 |
|  | BMI | 0.001 | 0.672 | 0.76 |
|  | ATG16L1 | 0.001 | 0.912 | 0.93 |
|  | **Disease phenotype * Age** | **0.044** | **0.005** | **0.03** |
| Interactions | NOD2 * ATG16L1 | 0.025 | 0.049 | 0.08 |
|  | ASA * BMI | 0.025 | 0.019 | 0.06 |
|  |  |  |  |  |
| **Category** | **FIRM. CLOSTRIDIUM GROUP XIVa** |  |  |  |
| **Sequencing** | **Sanger** | **R^2^** | **P value** | **FDR** |
| Main effects | **Disease phenotype** | **0.062** | **0.004** | **0.03** |
|  | *C. difficile* | 0.022 | 0.032 | 0.08 |
|  | NOD2 | 0.021 | 0.038 | 0.08 |
|  | Age | 0.000 | 0.812 | 0.87 |
|  | Gender | 0.007 | 0.201 | 0.28 |
|  | Smoking | 0.003 | 0.396 | 0.50 |
|  | Steroids | 0.001 | 0.581 | 0.68 |
|  | Immunomodulators | 0.002 | 0.490 | 0.560 |
|  | Anti -TNFα | 0.001 | 0.865 | 0.90 |
|  | BMI | 0.008 | 0.196 | 0.28 |
| Interactions | **Disease phenotype * Age** | **0.078** | **0.001** | **0.01** |
|  | **Anti-TNFα * *C. difficile*** | **0.059** | **0.005** | **0.03** |
|  | Smoking * Immunomodulators | 0.020 | 0.040 | 0.08 |
|  | Anti-TNFα * Gender | 0.037 | 0.026 | 0.07 |
|  | Steroids * BMI | 0.019 | 0.040 | 0.08 |
|  |  |  |  |  |
| **Sequencing** | **454 V1-V3** | **R^2^** | **P value** | **FDR** |
| Main effects | **Disease phenotype** | **0.052** | **0.001** | **0.01** |
|  | *C. difficile* | 0.020 | 0.036 | 0.08 |
|  | NOD2 | 0.022 | 0.03 | 0.08 |
|  | Age | 0.000 | 0.854 | 0.90 |
|  | Gender | 0.007 | 0.221 | 0.30 |
|  | Smoking | 0.003 | 0.388 | 0.49 |
|  | Steroids | 0.001 | 0.584 | 0.68 |
|  | Immunomodulators | 0.002 | 0.494 | 0.60 |
|  | Anti -TNFα | 0.013 | 0.245 | 0.33 |
|  | BMI | 0.008 | 0.165 | 0.24 |
| Interactions | **Disease phenotype * Age** | **0.088** | **0.003** | **0.02** |
|  | **Anti-TNFα * *C. difficile*** | **0.049** | **0.009** | **0.04** |
|  | Smoking * Immunomodulators | 0.020 | 0.034 | 0.08 |
|  | Anti-TNFα * Gender | 0.037 | 0.023 | 0.07 |
|  | Steroids * BMI | 0.019 | 0.038 | 0.08 |
|  |  |  |  |  |
| **Sequencing** | **454 V3-V5** | **R^2^** | **P value** | **FDR** |
| Main effects | Gender | 0.031 | 0.016 | 0.06 |
|  | Age | 0.020 | 0.05 | 0.09 |
|  | Steroids | 0.014 | 0.102 | 0.16 |
|  | Immunomodulators | 0.012 | 0.132 | 0.20 |
|  | BMI | 0.002 | 0.511 | 0.61 |
| Interactions | **Steroids * Immunomodulators** | **0.043** | **0.005** | **0.03** |
|  | **Steroids * BMI** | **0.034** | **0.008** | **0.04** |
|  |  |  |  |  |
| **Category** | **FIRM. BACILLUS** |  |  |  |
| **Sequencing** | **Sanger** | **R^2^** | **P value** | **FDR** |
| Main effects | **Disease phenotype** | **0.220** | **0.001** | **0.01** |
|  | *C. difficile* | 0.017 | 0.049 | 0.08 |
|  | Age | 0.007 | 0.210 | 0.29 |
|  | 5-ASA | 0.004 | 0.359 | 0.47 |
|  | Steroids | 0.000 | 0.861 | 0.90 |
|  | ATG16L1 | 0.016 | 0.169 | 0.24 |
| Interactions | **Disease phenotype * Steroids** | **0.037** | **0.004** | **0.03** |
|  | Disease phenotype * Age | 0.032 | 0.030 | 0.08 |
|  | 5-ASA * ATG16L1 | 0.028 | 0.037 | 0.08 |
|  |  |  |  |  |
| **Sequencing** | **454 V1-V3** | **R^2^** | **P value** | **FDR** |
| Main effects | **Disease phenotype** | **0.220** | **0.001** | **0.01** |
|  | *C. difficile* | 0.017 | 0.048 | 0.08 |
|  | Age | 0.006 | 0.228 | 0.31 |
|  | 5-ASA | 0.004 | 0.384 | 0.49 |
|  | Steroids | 0.000 | 0.841 | 0.89 |
|  | ATG16L1 | 0.018 | 0.144 | 0.21 |
| Interactions | **Disease phenotype * Steroids** | **0.037** | **0.005** | **0.03** |
|  | Disease phenotype * Age | 0.031 | 0.026 | 0.07 |
|  | 5-ASA * ATG16L1 | 0.030 | 0.034 | 0.08 |
|  |  |  |  |  |
| **Sequencing** | **454 V3-V5** | **R^2^** | **P value** | **FDR** |
| Main effects | **Disease phenotype** | **0132** | **0.001** | **0.01** |
|  | *C. difficile* | 0.023 | 0.036 | 0.08 |
|  | NOD2 | 0.024 | 0.019 | 0.06 |
|  | Steroids | 0.000 | 0.757 | 0.82 |
|  | Immunomodulators | 0.000 | 0.953 | 0.96 |
|  | ATG16L1 | 0.003 | 0.737 | 0.81 |
| Interactions | **Steroids * Immunomodulators** | **0.039** | **0.003** | **0.02** |
|  | NOD2 * ATG16L1 | 0.033 | 0.024 | 0.07 |
|  |  |  |  |  |
| **Category** | **PROTEOBACTERIA** |  |  |  |
| **Sequencing** | **Sanger** | **R^2^** | **P value** | **FDR** |
| Main effects | **Disease phenotype** | **0.058** | **0.005** | **0.03** |
|  | NOD2 | 0.024 | 0.035 | 0.08 |
|  | Gender | 0.022 | 0.038 | 0.08 |
|  |  |  |  |  |
| **Sequencing** | **454 V1-V3** | **R^2^** | **P value** | **FDR** |
| Main effects | **Disease phenotype** | **0.005** | **0.011** | **0.04** |
|  | NOD2 | 0.036 | 0.033 | 0.08 |
|  | Gender | 0.010 | 0.019 | 0.06 |
|  | 5-ASA | 0.008 | 0.08 | 0.12 |
|  | Steroids | 0.003 | 0.606 | 0.70 |
|  | Immunomodulators | 0.010 | 0.721 | 0.80 |
|  | Race | 0.002 | 0.615 | 0.70 |
| Interactions | Steroids * NOD2 | 0.069 | 0.027 | 0.08 |
|  | Steroids * Immunomodulators | 0.025 | 0.036 | 0.08 |
|  | 5-ASA * Race | 0.047 | 0.022 | 0.07 |
|  |  |  |  |  |
| **Sequencing** | **454 V3-V5** | **R^2^** | **P value** | **FDR** |
| Main effects | NOD2 | 0.027 | 0.025 | 0.07 |
|  | 5-ASA | 0.009 | 0.171 | 0.24 |
|  | Steroids | 0.004 | 0.373 | 0.48 |
|  | Race | 0.000 | 0.966 | 0.97 |
|  | ATG16L1 | 0.008 | 0.426 | 0.53 |
| Interactions | Steroids * NOD2 | 0.033 | 0.015 | 0.06 |
|  | 5-ASA * Race | 0.023 | 0.038 | 0.08 |
|  | NOD2 * ATG16L1 | 0.038 | 0.029 | 0.08 |
