# Supplementary material for: Inflammatory Bowel Diseases Phenotype, C. difficile and NOD2 Genotype Are Associated with Shifts in Human Ileum Associated Microbial Composition
Source: PLoS One. 2012 Jun 13;7(6):e26284. doi: 10.1371/journal.pone.0026284 (PMC3374607; doi:10.1371/journal.pone.0026284)
Supplement: Table S5 — Comparison between nonIBD control subjects with primary colon adenocarcinoma with non-IBD subjects without primary colon adenocarcinoma. Continuous variables (e.g. age, BMI, relative frequency of bacterial groups) were compared using the Wilcoxon rank sum test and categorical variables (e.g. genotype, smoking, race) were compared using the chi-square test. Note that none of the non-IBD control subjects had a positive C. difficile toxin or were taking any IBD medications. To address multiple comparison issues, the Benjamini-Hochberg method was applied to adjust P-values to the false discovery rate (FDR). Variables with FDR ≤0.05 are bolded. (DOCX) [file pone.0026284.s005.docx]

**Supplementary Table S5. Comparison between nonIBD control subjects with primary colon adenocarcinoma with non-IBD subjects without primary colon adenocarcinoma.** Continuous variables (e.g. age, BMI, relative frequency of bacterial groups) were compared using the Wilcoxon rank sum test and categorical variables (e.g. genotype, smoking, race) were compared using the chi-square test. Note that none of the non-IBD control subjects had a positive C. difficile toxin or were taking any IBD medications. To address multiple comparison issues, the Benjamini-Hochberg method was applied to adjust P-values to the false discovery rate (FDR). Variables with FDR ≤ 0.05 are bolded.

| **Variable** | **P-value** | **FDR** |
| --- | --- | --- |
| NOD2 composite genotype (NOD2^R/R^ + NOD2^R/NR^, NOD2^NR/NR^) | 0.13 | 0.3 |
| ATG16L1T300 A genotype (ATG16L1^R/R^, ATG16L1^R/NR^, ATG16L1^NR/NR^) | 0.48 | 0.68 |
| Gender (male, female) | 0.84 | 0.88 |
| Race (Caucasian, not Caucasian) | 0.64 | 0.84 |
| **Age of surgery** | **0.001** | **0.05** |
| Smoker (current, not current) | 0.88 | 0.88 |
| BMI | 0.77 | 0.88 |
|  |  |  |
| **Relative frequency phyla/subphyla categories (Sanger)** |  |  |
| Actinobacteria | 0.86 | 0.88 |
| Bacteroidetes | 0.76 | 0.88 |
| Firmicutes. Clostridium Group IV | 0.054 | 0.33 |
| Firmicutes Clostridium Group XIVa | 0.11 | 0.33 |
| Firmicutes Bacillus | 0.81 | 0.88 |
| Proteobacteria | 0.10 | 0.33 |
| Other Taxa | 0.40 | 0.67 |
|  |  |  |
| **Relative frequency phyla/subphyla categories (V1-V3)** |  |  |
| Actinobacteria | 0.44 | 0.67 |
| Bacteroidetes | 0.08 | 0.33 |
| Firmicutes. Clostridium Group IV | 0.06 | 0.33 |
| Firmicutes Clostridium Group XIVa | 0.42 | 0.67 |
| Firmicutes Bacillus | 0.07 | 0.33 |
| Proteobacteria | 0.65 | 0.84 |
| Other Taxa | 0.86 | 0.88 |
|  |  |  |
| **Relative frequency phyla/subphyla categories (V3-V5)** |  |  |
| Actinobacteria | 0.33 | 0.64 |
| Bacteroidetes | 0.28 | 0.58 |
| Firmicutes. Clostridium Group IV | 0.05 | 0.33 |
| Firmicutes Clostridium Group XIVa | 0.35 | 0.65 |
| Firmicutes Bacillus | 0.14 | 0.33 |
| Proteobacteria | 0.25 | 0.55 |
| Other Taxa | 0.45 | 0.67 |
|  |  |  |
| **Relative frequency of targeted microbial groups (qPCR)** |  |  |
| *C. coccoides-E. rectales* | 0.12 | 0.33 |
| *F. prausnitzii* | 0.14 | 0.33 |
