# Supplementary material for: Inflammatory Bowel Diseases Phenotype, C. difficile and NOD2 Genotype Are Associated with Shifts in Human Ileum Associated Microbial Composition
Source: PLoS One. 2012 Jun 13;7(6):e26284. doi: 10.1371/journal.pone.0026284 (PMC3374607; doi:10.1371/journal.pone.0026284)
Supplement: Table S6 — Distribution of the three common NOD2 genotypes in ileal CD, colitis and non-IBD control subjects. The three major NOD2 risk alleles that account for 80% of the NOD2 variants, are Leu1007fs (SNP13, rs2066847), R702W (SNP8, rs2066844), and G908W (SNP12, rs2066845). (DOCX) [file pone.0026284.s006.docx]

**Supplementary Table S6. Distribution of the three common NOD2 genotypes in ileal CD, colitis and non-IBD control subjects.** The three major NOD2 risk alleles that account for 80% of the NOD2 variants, are Leu1007fs (SNP13, rs2066847), R702W (SNP8, rs2066844), and G908W (SNP12, rs2066845).

| **Genotype** | **Ileal CD**  *(n = 52)* | **Colitis**  *(n = 58)* | **Control**  *(n = 60)* |
| --- | --- | --- | --- |
| **NOD2 homozygote and double heterozygote** | **9.6%** | **1.7%** | **0.0%** |
| Leu1007fs homozygote | 3.8% | 0.0% | 0.0% |
| Leu1007fs double heterozygote | 5.8% | 0.0% | 0.0% |
| **NOD2 heterozygote** | **28.8%** | **13.8%** | **11.7%** |
| Leu1007fs heterozygote | 13.5% | 5.2% | 0.0% |
| R702W heterozygote | 13.5% | 5.2% | 10.0% |
| G908W heterozygote | 1.8% | 3.4% | 1.7% |
