# Supplementary material for: Inflammatory Bowel Diseases Phenotype, C. difficile and NOD2 Genotype Are Associated with Shifts in Human Ileum Associated Microbial Composition
Source: PLoS One. 2012 Jun 13;7(6):e26284. doi: 10.1371/journal.pone.0026284 (PMC3374607; doi:10.1371/journal.pone.0026284)
Supplement: Methods S1 — (DOC) [file pone.0026284.s007.doc]

**Supplementary Methods**

***16S rRNA sequencing.***

*Sanger 16S rRNA primers.* Amplification of 16S rDNA was carried out using primers 27F (5’-AAGAGTTTGATCMTGGCTC-3’) and 1492R (5’-TACGGYTACCTTGTTACGACTT-3’). PCR fragments were subcloned into TOP TA pCR4.0, followed by transformation into *Escherichia coli* TOP10 (Invitrogen). Individual sub-clones were sequenced on 3730 XL Applied Biosystems capillary sequencer to a depth of 768 amplicons. The M13F (5’-TGTAAAACGACGGCCAGT-3'), M13R (5’-CAGGAAACAGCTATGACC-3') and 907R (5’-CCGTCAATTCCTTTRAGTTT-3') primers were used for sequencing.

*454 pyrosequencing primers.* The V1-V3 region was targeted by using barcoded primers 27F (5’-AAGAGTTTGATCMTGGCTC-3’) and 534R (5’-ATTACCGCGGCTGCTGG-3’), and the V3-V5 region was targeted by using barcoded primers 357F (5’- CCTACGGGAGGCAGCAG-3’) and 907R (5’CCGTCAATTCMTTTRAGT). These primers are identical to the primers used by the Human Microbiome Project to characterize the microbiota in healthy human subjects.

*Sequencing analysis software.* The software used for sequence analysis consisted of the function specific tools that were considered to be the standards in the academic community at the onset of this project. The analysis software pipeline accommodates reads produced by both Sanger and pyrosequencing platforms. Forward and reverse Sanger reads from individual clones were assembled using *phred* and *phrap* (Ewing and Green, 1998; Ewing et al., 1998) and sequences that did not form assemblages were discarded. Pyrosequences were sorted into libraries by barcode using *bartab* (Frank, 2009). All Sanger contigs and pyrosequences were screened for nucleotide quality (bases at 5’ and 3’ ends with mean Q <20 over a 10 nt window were discarded), ambiguous bases (sequences with >1 N were discarded), and minimum length (sequences <150 nt were discarded).

The *Infernal* RNA alignment tool (Nawrocki et al., 2009) was used to screen all sequences in terms of their fidelity to a Covariance Model (CM) derived from SSU rRNA secondary structure models provided by the lab of Dr. Robin Gutell (Cannone et al., 2002; Noller et al., 1986). Sequences that did not adequately match a bacterial CM model were removed from all subsequent analyses. This screening step aids in the process of eliminating non-rRNA sequences in general, and non-bacterial rRNA sequences in particular, ensuring compliance with the Health Insurance Portability and Accountability Act of 1996 (HIPAA) required for this project’s IRB. Chimera screening was performed by the tool *ChimeraSlayer* (Haas et al.), which requires that sequences be previously aligned with *NAST-iEr* (Haas et al.). Putative chimeras and other sequences that could not be aligned by *NAST-iEr* were removed from subsequent analyses.

Genera level taxonomic calls were produced by the *RDP Classifier* (Wang et al., 2007), which performs naïve Bayesian taxonomic classification versus a training set. This project used the code and training set provided by RDP on March 31, 2010 and April 6, 2010 respectively. Sequences with bootstrap scores below 50% were removed from subsequent analysis. Analyses available within this system, but not used for this data set include, BLAST (Altschul et al., 1990) against curated rRNA databases such as the Silva 104 (Pruesse et al., 2007) isolates), and the computation of often used ecological statistics (Magurran, 2003). The principal output of the pipeline was a tab-separated file that enumerated the number of times each unique taxonomy line was observed in each library. The final step also produces a summary of the processing activity that took place in each function specific tool. We note that as reported in a recent publication (Frank et al., 2011), the application of this pipeline to previously published sequence data produced nearly identical results to the original phylogenetic analysis.

***Statistical Methods.****Centered log ratio transformation*. Centered log ratio transformation (clr) projects a composition from the D-part Aitchison-simplex symmetrically to a Euclidian vector subspace for n subjects as listed below. In our case D = 7 and n =164, 164 and 169 respectively for Sanger, 454V1-V3, and 454V3-V5 datasets.

*clr* (*x_ij_*) = log (*x_ij_*) – mean(log *x_j_*), *i* = 1,2,…D-1, *j* = 1,2,….n

In other words, frequencies (0-1) are calculated first. To avoid 0 frequencies 0.5 are added to the raw counts for each bacterial category prior to calculating the frequencies. Then after calculating the logarithm of these adjusted frequencies, the composition within each observation (subject) is centered (Aitchison, 1986).

*Permutation based ANCOVA and MANCOVA (Anderson, 2001; McArdle and Anderson, 2001).* Generally, there are three advantages for this method: first, we do not have the multivariate normality restriction; second, distance calculation may not be of Euclidean (note that in our case, we still used Euclidean distance); third, it is able to handle high-dimensional dataset when *N < p*. The rationale for using Euclidean distance measurement as opposed to non-Euclidean dissimilarity measurements (e.g. Bray Curtiss) was to maintain consistency between the MANCOVA and the univariate analyses described below for each of the six individual bacterial categories. The principle of this method can be described as following:

The first steps for calculating F-values are the same as those in parametric method. The difference is how to derive p-values. Under the null hypothesis, there is no difference among groups (factors). In other words, if observations with different group labels are re-arranged, F-values will not change. Therefore, we can derive p-value from a permutation-based pseudo F-statistic: recalculate F values by randomly re-ordering observations w.r.t. group labels. If all possible shuffling are made, we can obtain the pseudo-F distribution under the null hypothesis and the corresponding p-value =$\frac{\text{(No. F}\geq\text{F}^{\text{*}})}{\text{(Total No. of F})}$, where F^*^ is the F-value for the original data. Finally, note that ANCOVA is just a special case for MANCOVA here.

*R codes for stepwise variable selection with permutation-based MANCOVA:*

##### Using Anderson's permutation-based MANOVA

library(vegan)

##### data should be cleaned first

X<-colnames(data)[21:(ncol(data)-1)]

X.pool<-X

X.pool

##### including all possible interaction terms second-order

for (i in 1:(length(X)-1)){

for (j in (i+1):length(X)){

X.pool<-c(X.pool,paste (X[i],X[j],sep=":"))

}

}

X.pool

##### variable selection

##### may loose the entry and leaving level to >0.05

p<-length(X.pool)

X.selected<-NULL

for (i in 1:p^2){

q<-length(X.pool)

pv<-rep(0,q)

### forward

for (j in 1:q){

### For categorical variable interaction term is always with main effects

temp<-unlist(strsplit(X.pool[j],":"))

if (length(temp)==1){

X.temp<-c(X.selected,temp)

}else{

X.temp<-c(X.selected,temp,X.pool[j])

}

X.temp<-unique(X.temp)

fit<-adonis(formula(paste("data[,2:7]~",paste(X.temp,collapse="+"))),data=data,method="euclidean")

### It is possible the tested term is an exact linear combination of others

indx<-(rownames(fit$aov.tab)==X.pool[j])

if (sum(indx)>0){

pv[j]<-fit$aov.tab[indx,6]

} else {

pv[j]<-1

}

}

if (min(pv)>=0.05) {

break

} else {

indx<-sort(pv,index.return=TRUE)$ix[1]

print(c("in",X.pool[indx],min(pv)))

X.selected<-c(X.selected,X.pool[indx])

X.pool<-X.pool[-indx]

}

### backward

### Be noticed that it is for "Type III" SS not the original SS in package as the orginal depends on the order of variables

for (k in 1:length(X.selected)){

X.temp<-X.selected[-k]

temp<-unlist(strsplit(X.selected[k],":"))

if (length(temp)==1){

X.temp<-c(X.temp,temp)

}else{

X.temp<-c(X.temp,temp,X.selected[k])

}

X.temp<-unique(X.temp)

fit<-adonis(formula(paste("data[,2:7]~",paste(X.temp,collapse="+"))),data=data,method="euclidean")

### Still it is possible the tested term is a exact linear combination of others

indx<-(rownames(fit$aov.tab)==X.selected[k])

if (sum(indx)>0){

pv<-fit$aov.tab[indx,6]

if (pv>=0.05) {

print(c("out",X.selected[k],pv))

X.pool<-c(X.pool,X.selected[k])

X.selected<-X.selected[-k]

break

}

}

if (sum(indx)==0){

print(c("out",X.selected[k],"not shown"))

X.pool<-c(X.pool,X.selected[k])

X.selected<-X.selected[-k]

break

}

}

}

X.selected

*Repeated measures ANOVA.* Repeated measures ANOVA follows the similar permutation steps as above (Anderson and Legendre,1999). The only difference is that permutations for repeated measures ANOVA are involved at two levels. It follows that the first level of permutation of observations to the combination of factors provides distribution against which to test the between-subject effects, while the second level of permutation within each subject provides distributions against which to test the within-subject (measures of three sequencing platforms) effect (Manly, 1997). Then *p* values are computed accordingly. R^2^ were computed from the repeated measures ANOVA by the equation R^2^= (Sum square of the variable) / (Total sum square). So for measurement, R^2^= (Sum square of measurements) / (Total sum square).

*R codes for permutation-based repeated measures ANOVA:*

btw.permutation <- function(data,f,vnum,varnames){

anova<-summary(aov(f,data = data))

print(anova)

names(anova) <- c("a","b")

ttt <- unlist(anova$a)

count <- vector(mode = "integer",length = vnum)

f0 <- vector(mode = "integer",length = vnum);

for (j in 1:vnum){

f0[j] <- ttt[paste("F value",j,sep = "")]

if (ttt[paste("Pr(>F)",j,sep = "")]<0.05) count[j]<- count[j]+1;

}

for (i in 1: 999){

ind <- rep(sample.int(157),3)

data.new <-data;

data.new[,colnames(data)==varnames] <- data[,colnames(data)==varnames][ind]

anova.new <- summary(aov(f,data = data.new))

names(anova.new) <- c("a","b")

xxx <- unlist(anova.new$a)

for (j in 1:vnum){

if (xxx[paste("F value",j,sep = "")]>f0[j]) count[j]<- count[j]+1;

}

}

print(varnames)

print(t(count/1000))

}

within.permutation <- function(data,f,vnum,varnames){

anova<-summary(aov(f,data = data))

print(anova)

names(anova) <- c("a","b")

ttt <- unlist(anova$b)

count <- 0

f0 <- ttt["F value1"]

if (ttt["Pr(>F)1"]<0.05) count<- count+1;

for (i in 1: 999){

mat <- c();

for (j in 1:157){

mat <- rbind(mat,sample(c(j,j+157,j+314)))

}

ind <- c(mat[,1],mat[,2],mat[,3])

data.new <-data;

data.new[,colnames(data)==varnames] <- data[,colnames(data)==varnames][ind]

anova.new <- summary(aov(f,data = data.new))

names(anova.new) <- c("a","b")

xxx <- unlist(anova.new$b)

if (xxx["F value1"]>f0) count<- count+1;

}

print(varnames)

print(t(count/1000))

}

############

btw.permutation(data,f,vnum,varnames)

############

within.permutation(data,f,vnum,varnames)

####Actino;

f <- as.formula("Act1 ~ Phenotype + Cdiff + steroids + BMI + NOD2 + smoking + AgeS + ATG16L1 + ASA + BMI:NOD2 + smoking:NOD2 + steroids:NOD2 + AgeS:ATG16L1 + ASA:Cdiff + Measure + Error(SUBJID)");

vnum <- 14;

varnames <- "Act1"

####Bac;

f <- as.formula("Bac1 ~ Phenotype + immunomodulator + SEX + smoking + NOD2 + ASA + AgeS + Cdiff + ATG16L1 + steroids + immunomodulator:SEX + smoking:NOD2 + Phenotype:ASA + ASA:AgeS + ASA:Cdiff + Cdiff:ATG16L1 + steroids:immunomodulator + ASA:NOD2 + Measure + Error(SUBJID)");

vnum <- 18;

varnames <- "Bac1"

####FirB1;

f <- as.formula("FirB1 ~ Phenotype + steroids + AgeS + ASA + ATG16L1 + Cdiff + NOD2 + immunomodulator + Phenotype:steroids + Phenotype:AgeS + ASA:ATG16L1 + steroids:immunomodulator + NOD2:ATG16L1 + Measure + Error(SUBJID)");

vnum <- 13;

varnames <- "FirB1"

####FirCL1;

f <- as.formula("FirCL1 ~ Phenotype + SEX + AgeS + smoking + Cdiff + NOD2 + ASA + BMI + ATG16L1 + Phenotype:AgeS + Cdiff:NOD2 + ASA:BMI + NOD2:ATG16L1+ Measure + Error(SUBJID)");

vnum <- 13;

varnames <- "FirCL1"

####FirCLa1;

f <- as.formula("FirCLa1 ~ Phenotype + AgeS + NOD2 + TNF + Cdiff + immunomodulator + smoking + SEX + steroids + BMI + Phenotype:AgeS + TNF:Cdiff + immunomodulator:smoking + TNF:SEX + steroids:BMI + steroids:immunomodulator + Measure + Error(SUBJID)");

vnum <- 16;

varnames <- "FirCLa1"

####Pro1;

f <- as.formula("Pro1 ~ NOD2 + Phenotype + SEX + steroids + immunomodulator + ASA + RACE + steroids:NOD2 + steroids:immunomodulator + ASA:RACE + NOD2:ATG16L1 + Measure + Error(SUBJID)");

vnum <- 11;

varnames <- "Pro1"

**References**

Aitchison J. (1986) The Statistical Analysis of Compositional Data, Monographs on Statistics and Applied Probability. Chapman & Hall Ltd., London (UK). 416p.

Aldhous MC, Drummond HE, Anderson N, et al. (2007)Does cigarette smoking influence the phenotype of Crohn’s disease? Analysis using the Montreal classification. Am J Gastroenterol. 102:577-88.

Altschul, S.F., Gish, W., Miller, W., Myers, E.W., and Lipman, D.J. (1990). Basic local alignment search tool. J Mol Biol *215*, 403-10.

Anderson, M.J. 2001. A new method for non-parametric multivariate analysis of variance. Austral Ecology, **26**: 32–46.

Anderson M. J. & Legendre P. (1999) An empirical comparison of permutation methods for tests of partial regression coefficients in a linear model. J. Stat. Comput. Simul. 62, 271 – 303.

Cannone, J.J., Subramanian, S., Schnare, M.N., Collett, J.R., D'Souza, L.M., Du, Y., Feng, B., Lin, N., Madabusi, L.V., Muller, K.M.*, et al.* (2002). The comparative RNA web (CRW) site: an online database of comparative sequence and structure information for ribosomal, intron, and other RNAs. BMC Bioinformatics *3*, 2.

Chen H, Lee A, Bowcock A et al. (2011) Influence of Crohn's disease risk alleles and smoking on disease location. Dis Colon Rectum. in press.

Cohen SH, Gerding DN, Johnson S, et al. (2007) Clinical practice guidelines for Clostridium difficile infection in adults: 2010 update by the society for healthcare epidemiology of America (SHEA) and the infectious diseases society of America (IDSA). Infect Control Hosp Epidemiol. 2010; 31:431-55.Ewing, B., and Green, P. (1998). Base-calling of automated sequencer traces using phred. II. Error probabilities. Genome Res *8*, 186-194.

Ewing, B., Hillier, L., Wendl, M.C., and Green, P. (1998). Base-calling of automated sequencer traces using phred. I. Accuracy assessment. Genome Res *8*, 175-185.

Frank, D.N., St Amand, A.L., Feldman, R.A., Boedeker, E.C., Harpaz, N., and Pace, N.R. (2007). Molecular-phylogenetic characterization of microbial community imbalances in human inflammatory bowel diseases. Proc Natl Acad Sci U S A *104*, 13780-13785.

Frank, D.N. (2009). BARCRAWL and BARTAB: Software tools for the design and implementation of barcoded primers for highly multiplexed DNA sequencing. BMC Bioinformatics *10*, 362.

Frank, D.N., Robertson, C.E., Hamm, C.M., Kpadeh, Z., Zhang, T., Chen, H., Zhu, W., Sartor, R.B., Boedeker, E.C., Harpaz, N.*, et al.* (2011). Disease phenotype and genotype are associated with shifts in intestinal-associated microbiota in inflammatory bowel diseases. Inflamm Bowel Dis *17*, 179-184.

Haas, B.J., Gevers, D., Earl, A.M., Feldgarden, M., Ward, D.V., Giannoukos, G., Ciulla, D., Tabbaa, D., Highlander, S.K., Sodergren, E.*, et al.* (2011) Chimeric 16S rRNA sequence formation and detection in Sanger and 454-pyrosequenced PCR amplicons. Genome Res *21*, 494-504.

Ley RE, Turnbaugh PJ, Klein S, Gordon JI. (2006) Microbial ecology: human gut microbes associated with obesity. Nature. 444:1022-3.

Magurran, A.E. (2003). Measuring Biological Diversity (Malden, USA, Blackwell).

Nawrocki, E.P., Kolbe, D.L., and Eddy, S.R. (2009). Infernal 1.0: inference of RNA alignments. Bioinformatics *25*, 1335-37.

Manly B. F. J. (1997) Randomization, Bootstrap and Monte Carlo Methods in Biology, 2nd ed. Chapman & Hall, London.

McArdle, B.H. and M.J. Anderson. 2001. Fitting multivariate models to community data: A comment on distance-based redundancy analysis. Ecology, **82**: 290–7

Nelson RL, Glenny AM, Song F. (2009) Antimicrobial prophylaxis for colorectal surgery. Cochrane Database Syst Rev. (1):CD001181

Noller, H.F., Asire, M., Barta, A., Douthwaite, S., Goldstein, T., Gutell, R.R., Moazed, D., Normanly, J., Prince, J., Stern, S.*, et al.* (1986). Structure, Function and Genetics of Ribosomes. In Structure, Function and Genetics of Ribosomes, H. Hardesty, and G. Kramer, eds. (Berlin, Springer Verlag).

Pruesse, E., Quast, C., Knittel, K., Fuchs, B., Ludwig, W., Peplies, J., and Glockner, F.O. (2007). SILVA: a comprehensive online resource for quality checked and aligned ribosomal RNA sequence data compatible with ARB. Nucleic Acids Res *35*, 7188-7196.

Unkart JT, Anderson L, Li E, et al. (2008) Risk factors for surgical recurrence after ileocolic resection of Crohn's disease. Dis Colon Rectum. 51:1211-6.

Wang, Q., Garrity, G.M., Tiedje, J.M., and Cole, J.R. (2007). Naive Bayesian classifier for rapid assignment of rRNA sequences into the new bacterial taxonomy. Appl Environ Microbiol *73*, 5261-5267.
